# The Burden of Acute Kidney Injury in Indian Pediatric Intensive Care Units

**DOI:** 10.5005/jp-journals-10071-23215

**Published:** 2019-08

**Authors:** Uma Ali

**Affiliations:** Division of Pediatric Nephrology, SRCC Children's Hospital, Mumbai, Maharashtra, India

## Abstract

**How to cite this article:** Ali U. The Burden of Acute Kidney Injury in Indian Pediatric Intensive Care Units. Indian J Crit Care Med 2019;23(8):349.

Acute kidney injury is a major game changer when it occurs in the setting of critical illness in children. It increases the risk of death independent of the severity of illness. In addition, one third of the AKI survivors carry the risk of future progression to chronic kidney disease. Zero deaths due to AKI by the year 2025 (0 by 25) is the ambitious global vision of the International Society of Nephrology. However, in many countries of the world including India we have not yet reached the starting post of understanding the total burden of AKI in children.

The burden of AKI could be higher in Indian children when compared to developed nations due to a higher incidence of infectious illnesses, wider use of both allopathic and non-allopathic nephrotoxic medications and the probability of having a higher subset of children born with lower nephron numbers secondary to premature births or intrauterine growth retardation that make them more vulnerable to AKI during critical illness.

The need of the hour is to have national or regional AKI prevalence studies to understand the extent of the problem and the regional differences in the predisposing conditions. Comparison of data is hampered by the prevalence of different methodologies for measuring serum creatinine and the use of different criteria for the diagnosis of AKI

As creatinine levels are physiologically low in infants and children due to lower muscle mass, it is important that serum creatinine is measured by enzymatic methods that measure true creatinine and avoid interference by other non-creatinine chromogens. Laboratories should validate their methodology by referencing to isotope dilution mass spectrometry (IDMS). This is already being done by some laboratories, but the standards need to become universal.

Several diagnostic criteria are currently used in pediatrics for defining AKI. The most common ones are the AKIN, the p RIFLE and the KDIGO criteria. The KDIGO does not need GFR calculations. It allows the use of either relative or absolute increases in serum creatinine and can be used for both adults and children. The uniform adoption of KDIGO criteria for defining AKI will allow valid comparison of data from different institutions.

There have been several small, single centre reports from different parts of India describing the prevalence and outcome of AKI in children. This issue carries an interesting snapshot from a PICU in Eastern UP where 50% of the cohort had viral encephalitis as the underlying illness.^1^ Until we are able to get the big picture, these snaphots from different parts of the country contribute important jigsaw pieces to complete the larger puzzle of the etiology and outcome of AKI in critically ill Indian children.
